# Label-free and washing-free alkaline phosphatase assay using a personal glucose meter

**DOI:** 10.1186/s13036-019-0182-3

**Published:** 2019-06-04

**Authors:** Jun Ki Ahn, Hyo Yong Kim, Chang Yeol Lee, Ki Soo Park, Hyun Gyu Park

**Affiliations:** 1 Department of Chemical and Biomolecular Engineering (BK21+ Program), KAIST, 291 Daehak-ro, Yuseong-gu, Daejeon, 34141 Republic of Korea; 2 Department of Biological Engineering, College of Engineering, Konkuk University, Seoul, 05029 Republic of Korea

**Keywords:** Alkaline phosphatase, Adenosine 5′-triphosphate, Personal glucose meter, Cascade enzymatic reaction, Biosensor

## Abstract

We herein describe a personal glucose meter (PGM)-based method for a label-free and washing-free determination of alkaline phosphatase (ALP) activity, which relies on the cascade enzymatic reactions promoted by hexokinase and pyruvate kinase to couple ALP activity with the amount of glucose. In principle, the presence of target ALP scavenges on adenosine 5′-triphosphate (ATP), a phosphate source for hexokinase-catalyzed reactions, and thus suppresses the ensuing cascade enzymatic reactions. As a result, the initial high amount of glucose is maintained and the amount of glucose, which is proportional to ALP activity, is simply measured by a hand-held PGM. Based on this novel strategy, we successfully determined the ALP activity down to 8.9 U/L with the high selectivity. In addition, the diagnostic capability of this method was demonstrated by reliably assaying the ALP activity in non-diluted human blood without any pretreatment steps.

**Electronic supplementary material:**

The online version of this article (10.1186/s13036-019-0182-3) contains supplementary material, which is available to authorized users.

## Background

A phosphatase is an enzyme that removes phosphate groups from its substrate by catalyzing the hydrolysis of a phosphomonoester and produces the molecule containing free hydroxyl groups. This enzyme is universally distributed in the cellular membrane and plays a critical role in the regulation of intracellular processes involved in cell cycle, growth, apoptosis, and signal transduction [1]. The most representative example in this type of enzyme is alkaline phosphatase (ALP), which is found in all tissues throughout the body, but is more concentrated on liver, bones, placenta, intestines, and kidneys [2]. Its abnormal level in serum is found to be closely related to various diseases and thus it has been used as a biomarker for the diagnosis of various diseases [3]. For examples, the low level of ALP in serum is often observed in malnourished patients, while the high level of ALP in serum is commonly associated with liver dysfunction, bone disease, prostate cancer, and bile-duct obstruction [4].

Up to date, various ALP assays have been developed, which rely on chromatographic, chemiluminescent, fluorescent, electrochemical, and surface enhanced Raman scattering signaling methods. However, most of them are not practically utilized due to their drawbacks such as the requirement for expensive reagents and specialized equipment. The ELISA method, which relies on the ALP-catalyzed conversion of *p*-nitrophenyl phosphate (*p*NPP) onto yellow colored products, is only used in the clinics to monitor ALP activity. A number of ELISA kits are commercially available on the market, but this method still has limitations such as the involvement of complicated operation, long assay time, and expensive equipment. Therefore, the development of novel strategies that operate in a stable, convenient, and cost-effective manner is highly required to replace ELISA-based ALP detection.

Towards this goal, we herein developed a simple method for a label-free and washing-free determination of ALP activity by employing a personal glucose meter (PGM), which has been regarded as one of the most successful point-of-care (POC) devices with a number of unique features including high portability, low cost, simple operation, and reliable quantitative ability [5]. Based on our recent finding that ATP was reliably determined in various real samples by linking the amount of ATP to glucose that is detectable by PGM, we designed a new PGM-based strategy for the determination of ALP activity [6]. With the developed system, we successfully analyzed the target ALP by simply measuring the PGM signal that is dependent on ALP activity, while overcoming the drawbacks in the conventional ALP assays.

## Methods

### Materials

D-glucose, magnesium chloride (MgCl_2_), Tris(hydroxymethyl)aminomethane hydrochloride (Tris-HCl), β-nicotinamide adenine dinucleotide phosphate hydrate (β-NADP), phosphoenolpyruvic acid (PEP), adenosine 5′-triphosphate disodium salt hydrate (ATP), alkaline phosphatase (ALP), human serum albumin (HSA), bovine serum albumin (BSA), trypsin, lysozyme, avidin, thrombin, hexokinase, glucose-6-phosphate dehydrogenase (G6PD), and pyruvate kinase were purchased from Sigma-Aldrich (St. Louis, MO, USA). Human blood was purchased from ZenBio Inc. (Research Triangle Park, NC, USA). All other chemicals were of analytical grade and used without further purification. Aqueous solutions were prepared using ultrapure DNase/RNase-free distilled water (D.W.) purchased from Bioneer®. The personal glucose meter (PGM) from *Accu-Chek Active* (Roche, Basel, Switzerland) was used in this work.

### Alkaline phosphatase assay using a PGM

16 μL of ALP at various concentrations or HSA, BSA, trypsin, lysozyme, avidin, and thrombin (2.5 uM) was first mixed with 2 μL of ATP (50 mM) and 2 μL of 10X reaction buffer (500 mM Tris-HCl, 10 mM MgCl_2_, pH 7.4) and incubated at 37 °C for 30 min. Next, the above ALP reaction solution was mixed with 5 μL of D-glucose (50 mM), 1 μL of β-NADP (50 mM), 1 μL of PEP (100 mM), and 5 μL of 10X reaction buffer (1 M Tris-HCl, 100 mM MgCl_2_, pH 7.4), which was then incubated with 18 μL of enzyme mixture containing hexokinase (5 U), pyruvate kinase (5 U), and G6PD (0.4 U) at 30 °C for 30 min. Finally, the resulting glucose level in the mixture was measured by a PGM.

### Recovery test

The target ALP at different concentrations were spiked into non-diluted human blood, which was directly analyzed by following the same detection procedure used in the buffer solution. To determine the amount of spiked ALP, the calibration curve was first drawn with a set of standards containing a known amount of ALP in the non-diluted human blood and the unknown amount of ALP was then determined based on the calibration curve.

## Results and discussion

### Detection principle of the PGM-based ALP assay

**Fig. 1:**
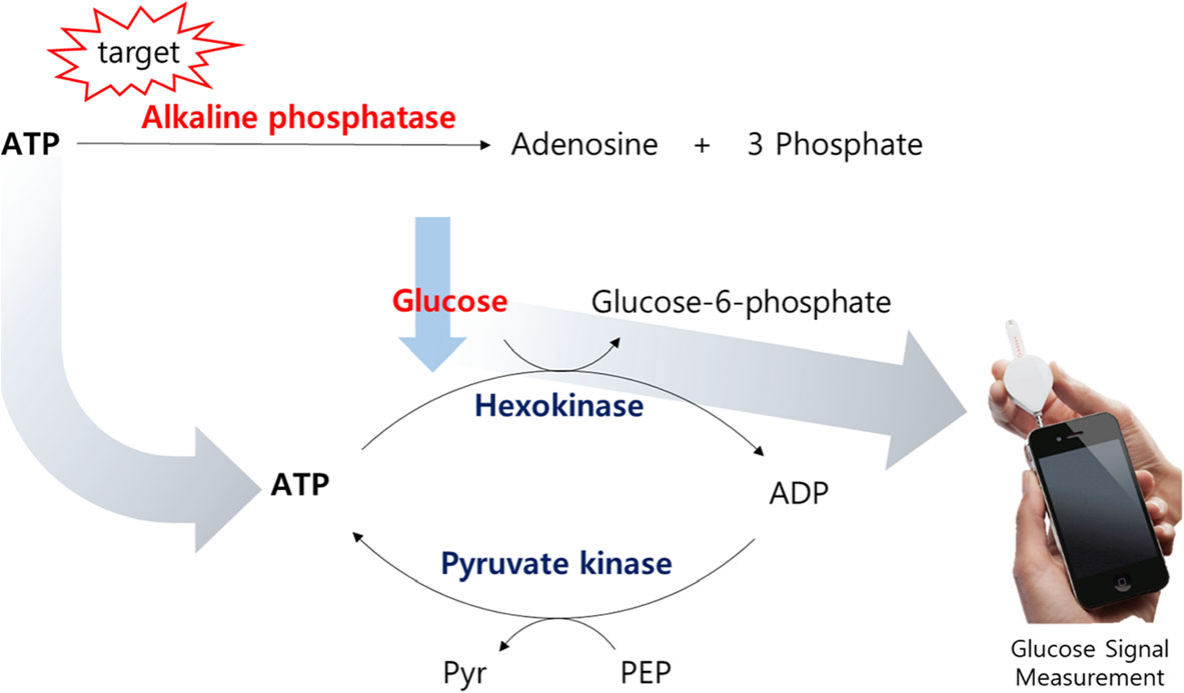
Schematic illustration of the PGM-based ALP assay (ATP: adenosine 5′-triphosphate, ADP: adenosine 5′-diphosphate, PEP: phosphoenolpyruvic acid, Pyr: Pyruvate)

The conceptual design of the PGM-based ALP assay is illustrated in Fig. 1. The proposed system relies on the cascade enzymatic reactions promoted by hexokinase and pyruvate kinase that link the amount of ATP to glucose [6]. In the absence of target ALP, the intact ATP enables hexokinase to catalyze the conversion of glucose to glucose-6-phosphate by providing a phosphate group to glucose and accordingly the amount of glucose is decreased. In addition, the adenosine 5′-diphosphate (ADP), which is generated after the hexokinase-catalyzed enzymatic reaction, is recovered to ATP by pyruvate kinase that catalyzes the conversion of phosphoenolpyruvic acid (PEP) to pyruvate. The regenerated ATP is again supplemented to catalyze multiple rounds of cascade enzymatic reactions, resulting in a significantly decreased amount of glucose. On the other hand, the presence of ALP scavenges on ATP, which suppresses the subsequent, cascade enzymatic reactions promoted by hexokinase and pyruvate kinase. Notably, ALP shows the best enzymatic activity at pH values from 8 to 10, at which other enzymes such as hexokinase, pyruvate kinase, and G6PD can hardly resist. Thus, we selected pH 7.4 at which other enzymes work fine and ALP activity for the hydrolysis of ATP is slightly reduced to ca. 80% [7]. Under these conditions, we can successfully monitor the ALP activity using a PGM despite the slight sacrifice of ALP activity. As a result, the initial high amount of glucose is retained and the amount of glucose that is proportional to ALP activity is simply monitored by a hand-held PGM.

### Detection feasibility of the PGM-based ALP assay

**Fig. 2:**
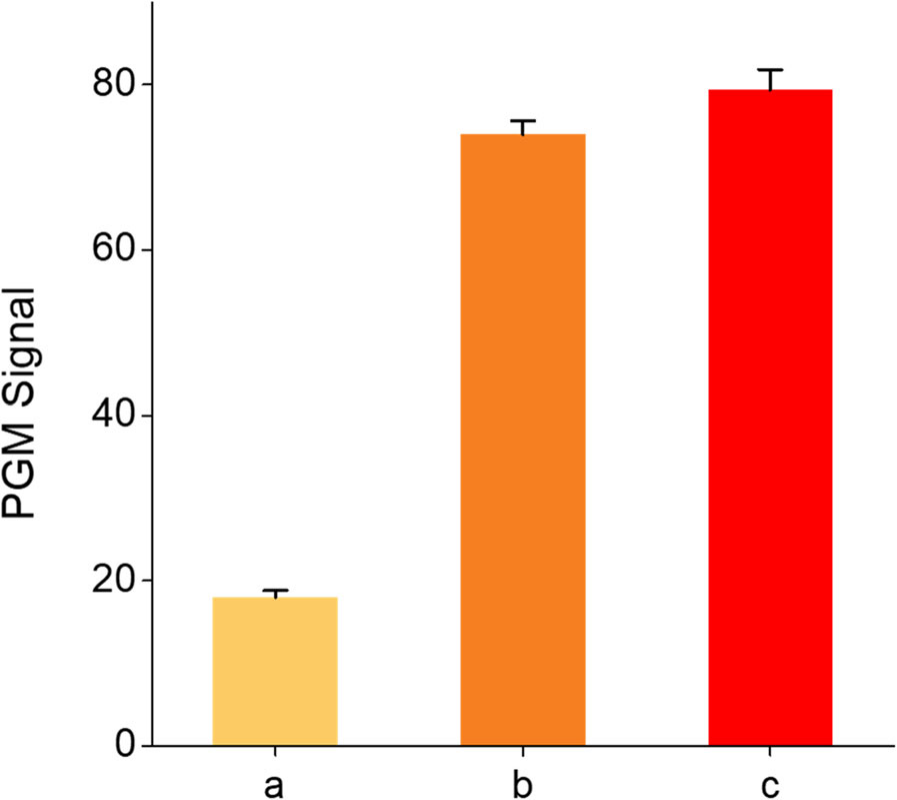
Detection feasibility of the PGM-based ALP assay. The PGM signals from different samples containing ATP only (**a**), both ALP and ATP (**b**), and neither ALP nor ATP (**c**). The concentrations of ALP and ATP were 400 U/L and 1 mM, respectively

First, the detection feasibility of the PGM-based ALP assay was validated by measuring PGM signals in different samples. As shown in Fig. 2, the significantly reduced PGM signal was observed in the absence of target ALP because the intact ATP initiated the cascade enzymatic reactions promoted by hexokinase and pyruvate kinase, resulting in the decrease of glucose amount (Fig. 2(a)). However, in the presence of target ALP, the ATP was wasted and the subsequent cascade enzymatic reactions were not executed, and thus the high PGM signal was obtained (Fig. 2(b)), which was comparable to the one of the negative control in which both ALP and ATP were excluded (Fig. 2(c)). These observations clearly confirm that ALP that utilizes ATP as the substrate regulates the cascade enzymatic reactions and the resulting changes of glucose amount are simply monitored by a hand-held PGM. Next, the optimal conditions for the efficient analysis of ALP activity were also investigated. The results of experiments in which the ATP amount and ALP reaction time were varied demonstrate that 5 mM of ATP and 30 min of ALP reaction were ideal to achieve the best detection performance, which were used for further experiments (Additional file 1: Figure S1).

### Selectivity of the PGM-based ALP assay

**Fig. 3:**
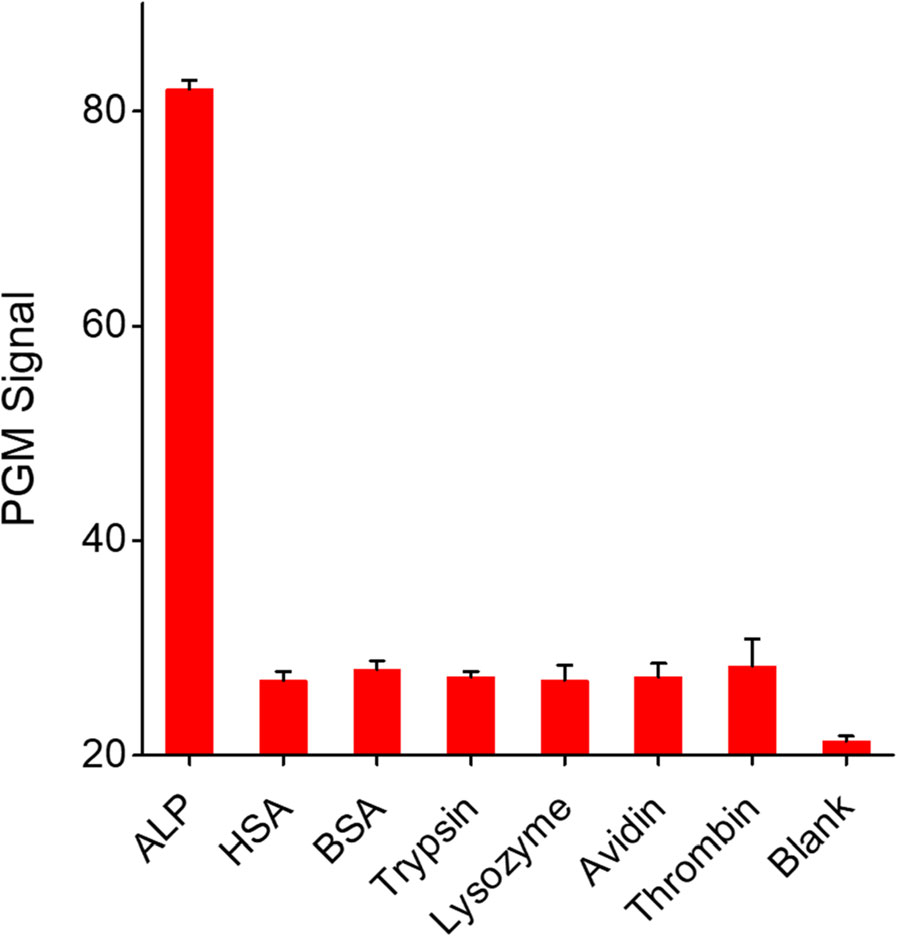
Detection selectivity of the PGM-based ALP assay. The concentrations of ALP and other proteins (HSA, BSA, trypsin, lysozyme, avidin, and thrombin) were 600 U/L (0.5 μM) and 2 μM, respectively

The selectivity of the PGM-based ALP assay was investigated by examining the abilities of interfering proteins such as BSA, HSA, trypsin, lysozyme, avidin, and thrombin to change the PGM signal. As presented in Fig. 3, the high PGM signal was only observed from the sample containing the ALP. On the other hand, the presence of interfering proteins produced the negligible PGM signal even though their concentrations were at four times higher than that of ALP. These results clearly confirm the high selectivity of our ALP detection method.

### Sensitivity of the PGM-based ALP assay

**Fig. 4:**
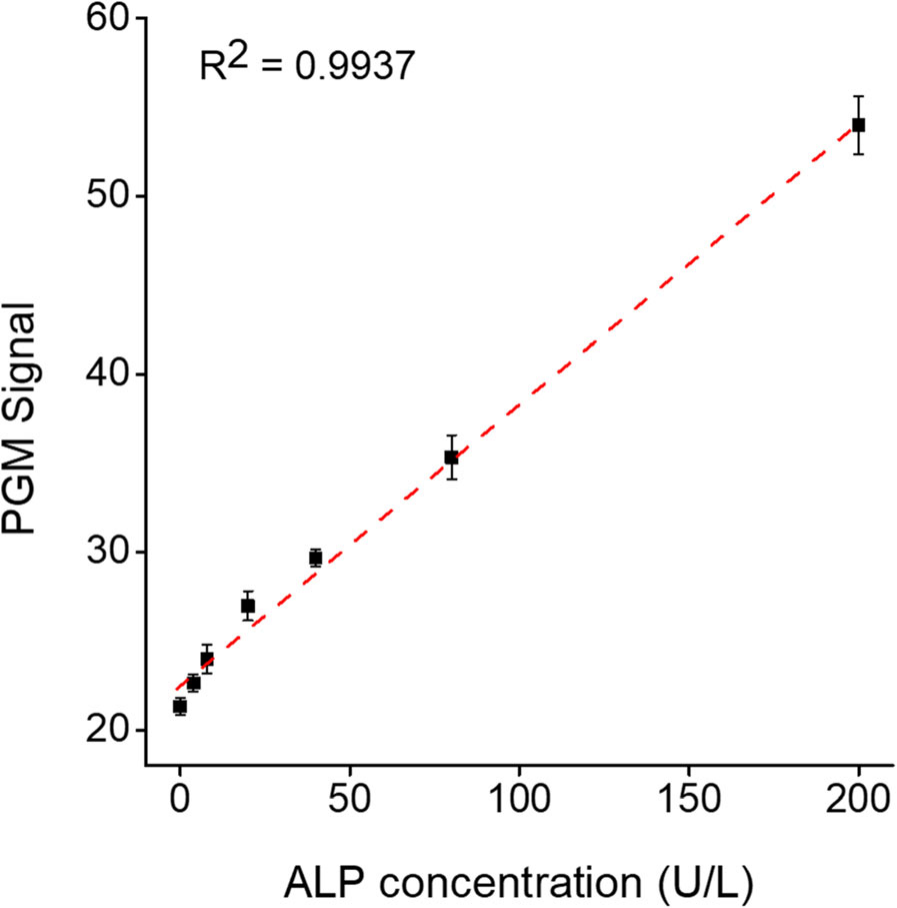
Detection sensitivity of the PGM-based ALP assay. The PGM signal was measured as a function of ALP concentration

The sensitivity of the PGM-based ALP assay was determined by measuring the PGM signal as a function of ALP concentration. The results in Fig. 4 show that the PGM signal increases with increasing concentration of ALP. An excellent linear relationship (R^2^ = 0.9937) existed in the range from 8 to 200 U/L and the limit of detection (LOD) (3σ/slope) was ca. 8.9 U/L (7.4 pM), which is comparable or higher than those from other ALP assay methods (Additional file 1: Table S1). However, it should be noted that the detection sensitivity of our strategy is good enough to cover the clinically important concentration of ALP in human blood (44–147 U/L) [8]. In addition, the developed method is simply operated by a hand-held PGM without the requirement of bulky operation system and expensive reagents.

### Practical applicability of the PGM-based ALP assay

**Fig. 5:**
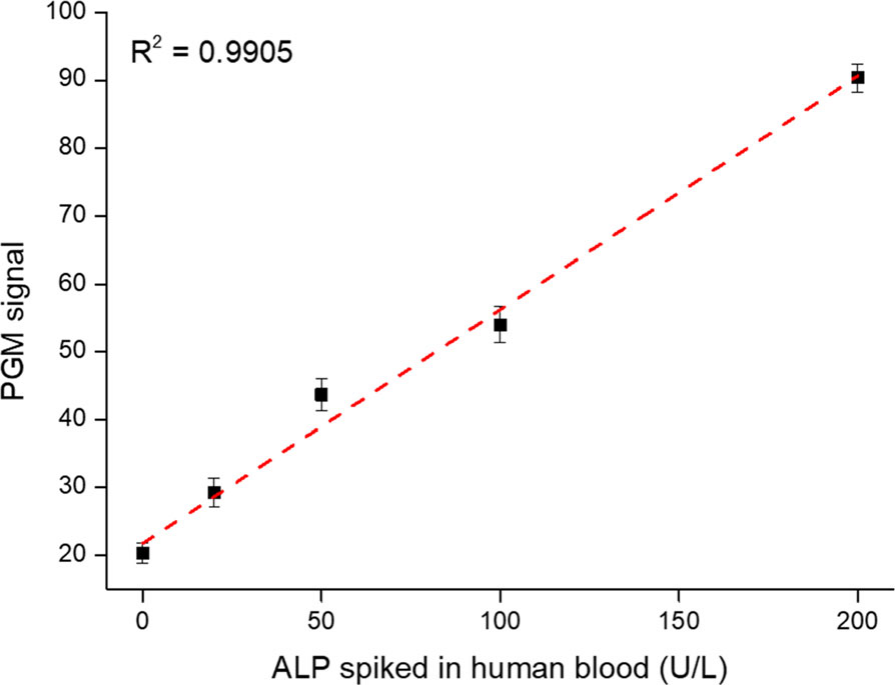
The PGM signal as a function of ALP spiked in non-diluted human blood

Finally, the practical applicability of the PGM-based ALP assay was demonstrated by analyzing ALP present in non-diluted human blood. As shown in Fig. 5, ALP activity in the human blood was determined based on the standard addition method [9]. The results demonstrate that the PGM signal increased with increasing concentration of ALP spiked into human blood in the range from 0 to 200 U/L and the ALP activity present in the human blood was determined to be 66 U/L which is in good agreement with the typical ALP activity in the human blood [8]. Furthermore, the excellent reproducibility and precision of our strategy was confirmed by a coefficient of variation (CV) less than 9% and a recovery ratio between 98 and 107% (Table 1). Importantly, the ALP in human blood was analyzed without either dilution or pre-treatment steps, which is normally executed in most ALP methods to avoid the signal interference [10]. Overall, these results prove that the developed ALP system could be utilized to reliably determine the ALP activity in human blood and applied to the assay of ALP contained in the whole blood samples obtained by finger prick, ensuring its future clinical application.

**Table 1:** Determination of ALP spiked into non-diluted human blood ^a^

ALP in human blood (U/L)	Added ALP (U/L)	Measured ALP (U/L)^b^	SD^c^	CV (%)^d^	Recovery (%) ^e^
66	30	101.94	8.85	8.68	106.19
	50	113.74	5.11	4.49	98.05
	100	168.82	9.01	5.34	101.70

^a^To determine the concentration of ALP in human blood, a calibration curve was first created by using standards having known concentrations of ALP in human blood (Fig. 5). Based on this calibration curve, the PGM signals from unknown samples were used to determine the concentrations of ALP in human blood. ^b^Mean of three measurements. ^c^ Standard deviation of three measurements. ^d^Coefficient of variation = SD/mean × 100. ^e^Measured value/added value × 100

## Conclusions

We herein developed a PGM-based method for a label-free and washing-free ALP assay. This strategy relies on the working principle that ALP, which scavenges on ATP and suppresses the subsequent cascade enzymatic reactions, changes the amount of glucose that is simply measured by a hand-held PGM. With this design principle, the target ALP was successfully determined with high selectivity even in human blood. Importantly, the developed system was simply operated with a PGM, while overcoming the drawbacks in the previous ALP assays that require expensive reagents and specialized equipment. In addition, the developed ALP assay utilizes a PGM that is commercially available on the market at the low cost and thus it would be readily applied in a various POC tests. Finally, we believe that the developed system would be practically used in the clinics and pave the way for the new enzymatic assay.

## Additional file

Additional file 1: **Table S1**. Summary of the previously reported ALP assay methods. **Figure S1.** Optimization of (a) ATP concentration and (b) ALP reaction time. P_0_ and P are defined as PGM signals in the absence and presence of ALP (200 U/L), respectively. (DOCX 191 kb)

## Data Availability

All data generated or analyzed during this study are included in this published article [and its supplementary information files].

## Abbreviations

ALP: alkaline phosphatase
ATP: adenosine 5′-triphosphate
BSA: bovine serum albumin
G6PD: glucose-6-phosphate dehydrogenase
HSA: human serum albumin
PEP: phosphoenolpyruvic acid
PGM: personal glucose meter
Tris-HCl: Tris(hydroxymethyl)aminomethane hydrochloride
β-NADP: β-nicotinamide adenine dinucleotide phosphate hydrate
