# Supplementary material for: Label-free and washing-free alkaline phosphatase assay using a personal glucose meter
Source: J Biol Eng. 2019 Jun 4;13:51. doi: 10.1186/s13036-019-0182-3 (PMC6551882; doi:10.1186/s13036-019-0182-3)
Supplement: Supplementary file 1 — Table S1. Summary of the previously reported ALP assay methods. Figure S1. Optimization of (a) ATP concentration and (b) ALP reaction time. P0 and P are defined as PGM signals in the absence and presence of ALP (200 U/L), respectively. (DOCX 191 kb) [file 13036_2019_182_MOESM1_ESM.docx]

**Additional File**

**Label-free and washing-free alkaline phosphatase assay using a personal glucose meter**

Jun Ki Ahn,^†a^ Hyo Yong Kim,^†a^ Chang Yeol Lee,^a^ Ki Soo Park^*b^ and Hyun Gyu Park^*a^

^a^ Department of Chemical and Biomolecular Engineering (BK21+ Program), KAIST, 291 Daehak-ro, Yuseong-gu, Daejeon 34141, Republic of Korea.

^b^ Department of Biological Engineering, College of Engineering, Konkuk University, Seoul 05029, Republic of Korea.

*Corresponding authors.

E-mail: hgpark@kaist.ac.kr (H.G. Park); Phone: +82-42-350-3932; Fax: +82-42-350-3910.

E-mail: kskonkuk@gmail.com (K.S. Park); Phone: +82-2-450-3742; Fax: +82-2-450-3742.

^†^ These authors equally contributed to this work.

**Table S1.** Summary of the previously reported ALP assay methods.

| **Key elements** | **Detection method** | **Detection limit** | **Sample** | **Reference** |
| --- | --- | --- | --- | --- |
| β-cyclodextrin-modified quantum dots | Fluorescence | 10 U/L | - | [1] |
| dsDNA-templated copper nanoparticles | Fluorescence | 0.3 U/L | Diluted serum | [2] |
| Gold nanoparticles | Colorimetry | 1000 U/L | Serum | [3] |
| Peroxidase activity of copper ions | Fluorescence and colorimetry | 4.3 pM | Diluted serum | [4] |
| Carbon quantum dots | Fluorescence | 1.4 U/L | - | [5] |
| Molybdophosphate | Electrochemistry | 0.5 U/L | - | [6] |
| Graphitic carbon nitride nanosheet | Fluorescence | 0.08 U/L | Diluted serum | [7] |
| Microfluidic paper-based analytical device (μPAD) | Flow length measurement | 75 U/L | Diluted serum | [8] |
| Lab-on-a-chip (LOC) | Fluorescence | 0.1 U/L | Serum | [9] |
| Personal glucose meter (PGM) | Electrochemistry | 8.9 U/L  (7.4 pM) | Blood | This work |


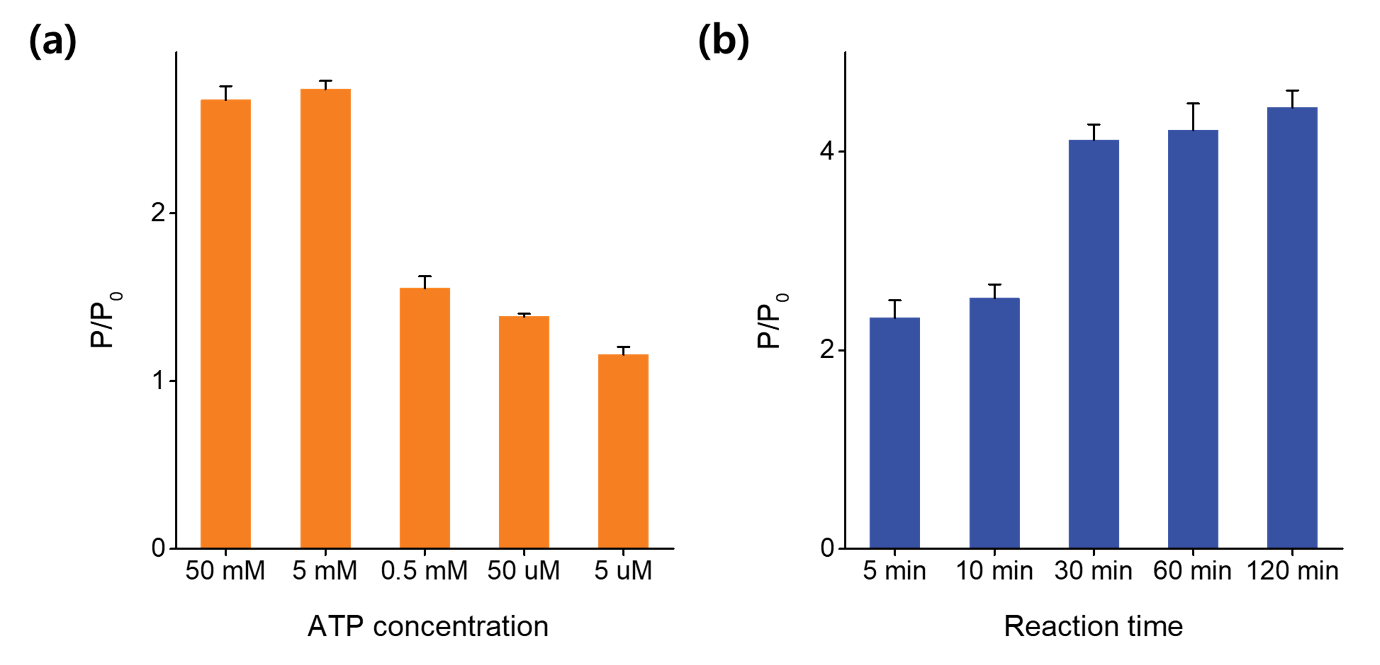
**Figure S1.** Optimization of (a) ATP concentration and (b) ALP reaction time. P_0_ and P are defined as PGM signals in the absence and presence of ALP (200 U/L), respectively.

**References**

1. Jia L, Xu JP, Li D, Pang SP, Fang Y, Song ZG, et al. Fluorescence detection of alkaline phosphatase activity with β-cyclodextrin-modified quantum dots. Chem Commun. 2010;46:7166-8.
2. Zhang L, Zhao J, Duan M, Zhang H, Jiang J, Yu R. Inhibition of dsDNA-templated copper nanoparticles by pyrophosphate as a label-free fluorescent strategy for alkaline phosphatase assay. Anal Chem. 2013;85:3797-801.
3. Li CM, Zhen SJ, Wang J, Li YF, Huang CZ. A gold nanoparticles-based colorimetric assay for alkaline phosphatase detection with tunable dynamic range. Biosens Bioelectron. 2013;43:366-71.
4. Park KS, Lee CY, Park HG. A sensitive dual colorimetric and fluorescence system for assaying the activity of alkaline phosphatase that relies on pyrophosphate inhibition of the peroxidase activity of copper ions. Analyst. 2014;139:4691-5.
5. Qian Z, Chai L, Tang C, Huang Y, Chen J, Feng H. Carbon quantum dots-based recyclable real-time fluorescence assay for alkaline phosphatase with adenosine triphosphate as substrate. Anal Chem. 2015;87:2966-73.
6. Shen C, Li X, Rasooly A, Guo L, Zhang K, Yang M. A single electrochemical biosensor for detecting the activity and inhibition of both protein kinase and alkaline phosphatase based on phosphate ions induced deposition of redox precipitates. Biosens Bioelectron. 2016;85:220-5.
7. Xiang MH, Liu JW, Li N, Tang H, Yu RQ, Jiang JH. A fluorescent graphitic carbon nitride nanosheet biosensor for highly sensitive, label-free detection of alkaline phosphatase. Nanoscale. 2016;8:4727-32.
8. Zhang L, Nie J, Wang H, Yang J, Wang B, Zhang Y, et al. Instrument-free quantitative detection of alkaline phosphatase using paper-based devices. Anal Methods. 2017;9:3375-9.
9. Cao XY, Kong FZ, Zhang Q, Liu WW, Liu XP, Li GQ, et al. iPhone-imaged and cell-powered electrophoresis titration chip for the alkaline phosphatase assay in serum by the moving reaction boundary. Lab Chip. 2018;18:1758-66.
